# Heartland Virus in Lone Star Ticks, Alabama, USA

**DOI:** 10.3201/eid2608.200494

**Published:** 2020-08

**Authors:** Brent C. Newman, William B. Sutton, Abelardo C. Moncayo, Holly R. Hughes, Ali Taheri, Thomas C. Moore, Callie J. Schweitzer, Yong Wang

**Affiliations:** Tennessee State University, Nashville, Tennessee, USA (B.C. Newman, W.B. Sutton, A. Taheri);; Tennessee Department of Health, Nashville (A.C. Moncayo, T.C. Moore);; Centers for Disease Control and Prevention, Fort Collins, Colorado, USA (H.R. Hughes);; US Department of Agriculture, Huntsville, Alabama, USA (C.J. Schweitzer); Alab; ma Agricultural and Mechanical University, Huntsville (Y. Wang)

**Keywords:** HRTV, lone star tick, southeastern United States, surveillance, tick-borne illness, vector-borne infections, viruses, Heartland virus, Amblyomma americanum, Alabama, phleboviruses

## Abstract

We detected Heartland virus (HRTV) in lone star nymphs collected in 2018 in northern Alabama, USA. Real-time reverse transcription PCR selective for the small segment of the HRTV genome and confirmatory sequencing of positive samples showed high identity with HRTV strains sequenced from Tennessee and Missouri.

Heartland virus (HRTV) is an emerging pathogenic phlebovirus first identified in the United States in 2009 and now reported in 15 states (*1*,*2*)*.* Nymphal lone star ticks (*Amblyomma americanum*) are considered the primary vectors of HRTV, and a variety of domestic and endemic mammalian species are potential amplification hosts of this virus (*2*,*3*). Although *A. americanum* ticks are well-established throughout the eastern, southeastern, and midwestern United States, their range is expanding northward and westward, most likely because of increased host availability and abundance, changes in environmental and climatic conditions, and adaptive genetic variation (Figure, panel A) (*4*). We tested for HRTV in *A. americanum* ticks collected in Alabama, USA, a state within the range of this vector where HRTV has not been documented previously from ticks.

**Figure:**
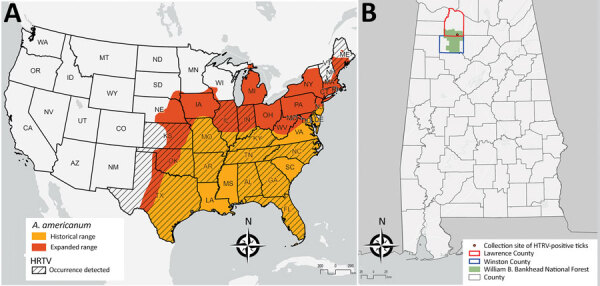
Distribution of HRTV and range of *Amblyomma americanum* ticks. A) Geographic distribution of Heartland virus, United States, 2009–2020 (*1*,*2*) with historical and expanded range of *A. americanum* ticks adapted from (*4*). B) Location of the William B. Bankhead National Forest within Lawrence and Winston Counties, Alabama, and collection site of the HRTV-positive *A. americanum* nymphs. All maps were created by using ArcGIS Pro 2.5 (ESRI, https://www.esri.com/en-us/home). HRTV, Heartland virus.

From June 1, 2018, through August 31, 2018, we collected ticks as previously described (*5*) in the William B. Bankhead National Forest, Alabama (34.2270°N, 87.3461°W; Figure, panel B). In preparation for pathogen screening, we separated ticks into pools. Nymph tick pools ranged from 1 to 5 tick(s) of the same species per pool. We screened adult ticks individually (i.e., 1 adult tick per pool) (Appendix Table). We did not include larvae in pathogen screening. We used molecular methods to extract viral RNA and detect the small (S) segment of the HRTV genome using the HRTV-4 primer and probe set (*6*) in tick pools (Appendix Table). We sequenced HRTV-4–positive samples using the Ion Torrent Personal Genomic Machine system (Life Technologies, https://www.thermofisher.com) at the Centers for Disease Control and Prevention (CDC; Fort Collins, CO, USA) as described previously (*7*). We obtained sequences of the HTRV S segment of other HRTV samples and strains from the GenBank database, and aligned sequences using the MUSCLE alignment tool (https://www.ebi.ac.uk/Tools/msa/muscle) in MEGA software (*8*). We also included a closely related severe fever with thrombocytopenia syndrome virus isolate from the GenBank database as an outgroup for this analysis. We used a maximum-likelihood tree approach with 1,000 bootstrap replications to generate the genetic relationships between the Alabama samples and the other HRTV samples available through the GenBank database. 

We collected 964 ticks, of which 921 were *A. americanum* (872 nymphs, 22 adult males, and 27 adult females) and 43 were *Dermacentor variabilis* (20 adult males and 23 adult females). We tested the ticks in 337 screened tick pools (Appendix Table). We amplified HRTV-4 from 5 pools that each contained 4 *A. americanum* nymphs. Therefore, the bias-corrected maximum-likelihood estimate of the infection rate (*9*) in questing *A. americanum* nymphs collected from the William B. Bankhead National Forest during 2018 was 0.58 (95% CI 0.21–1.27) and minimum infection rate (*9*) was 0.57 (95% CI 0.07–1.07) per 100 ticks screened on the basis of 235 nymph pools tested. To confirm results, we randomly selected homogenate from 3 of 5 HRTV-4–positive pools and submitted 3 individual RNA samples for sequencing at CDC. Sequencing RNA directly from tick homogenate confirmed HRTV in each of the 3 pools. Although we did not obtain whole-genome sequences, we identified partial coding sequences of all 3 HRTV segments in each pool. Maximum-likelihood phylogenetic inference of 730 nt of the S segment confirmed the BLAST analysis (https://blast.ncbi.nlm.nih.gov/Blast.cgi) and placed the generated HRTV S segment (submitted under GenBank accession no. MT052710) in a well-supported clade with HRTV strains previously described in Missouri and Tennessee (Appendix Figure).

Our findings of HRTV in *A. americanum* ticks in Alabama update knowledge of the virus’ distribution in the United States (Figure, panel A). Our findings also suggest *A. americanum* nymphs are the primary vectors of HRTV. As the geographic range of *A. americanum* continues to expand, we encourage enhanced surveillance and screening for HRTV to provide a more accurate and up-to-date understanding of where this tickborne virus probably occurs in the United States. Treatment for HRTV infection is limited to supportive care only; clinical data from the southeastern United States show that Heartland virus has a 10% death rate (*10*). Surveillance of HRTV in tick vector species is necessary to gain a comprehensive understanding of the environmental determinants that may put humans at risk for encountering the vector and to identify the geographic host range (both current and potential) of this emerging pathogen in the United States.

Appendix Additional information about ticks and polygenetic analysis of Heartland virus, Alabama, USA.
