# Supplementary material for: Heartland Virus in Lone Star Ticks, Alabama, USA
Source: Emerg Infect Dis. 2020 Aug;26(8):1954–6. doi: 10.3201/eid2608.200494 (PMC7392462; doi:10.3201/eid2608.200494)
Supplement: Appendix — Additional information about ticks and polygenetic analysis of Heartland virus, Alabama, USA. [file 20-0494-Techapp-s1.pdf]

Article DOI: https://doi.org/10.3201/eid2608.200464

# Heartland Virus in Lone Star Ticks, Alabama, USA

## Appendix

**Appendix Table.** Total number of questing *Amblyomma americanum* and *Dermacentor variabilis* ticks collected at the William B. Bankhead National Forest, Alabama, June–August 2018

| | | | Pools screened and no. per pool | | | | |
|---|---|---|---|---|---|---|---|
| Species | Stage, sex | No. screened | 1 | 2 | 3 | 4 | 5 |
| *A. americanum* | Adult, F | 27 | 27 | 0 | 0 | 0 | 0 |
| | Adult, M | 22 | 22 | 0 | 0 | 0 | 0 |
| | Nymph | 872 | 1 | 9 | 71 | 130 | 24 |
| Subtotal | | 921 | 50* | 18 | 213 | 520 | 120 |
| *D. variabilis* | Adult, F | 23 | 23 | 0 | 0 | 0 | 0 |
| | Adult, M | 20 | 20 | 0 | 0 | 0 | 0 |
| Subtotal | | 53 | 53 | 0 | 0 | 0 | 0 |
| Total | Adult | 92 | 92 | 0 | 0 | 0 | 0 |
| | Adult, F | 50 | 50 | 0 | 0 | 0 | 0 |
| | Adult, M | 42 | 42 | 0 | 0 | 0 | 0 |
| | Nymph | 872 | 1 | 9 | 71 | 130 | 24 |
| | | ∑ = 964 | | | | | |

*Calculated as (no. per pool × pools screened) + column total of no. per pool by species.

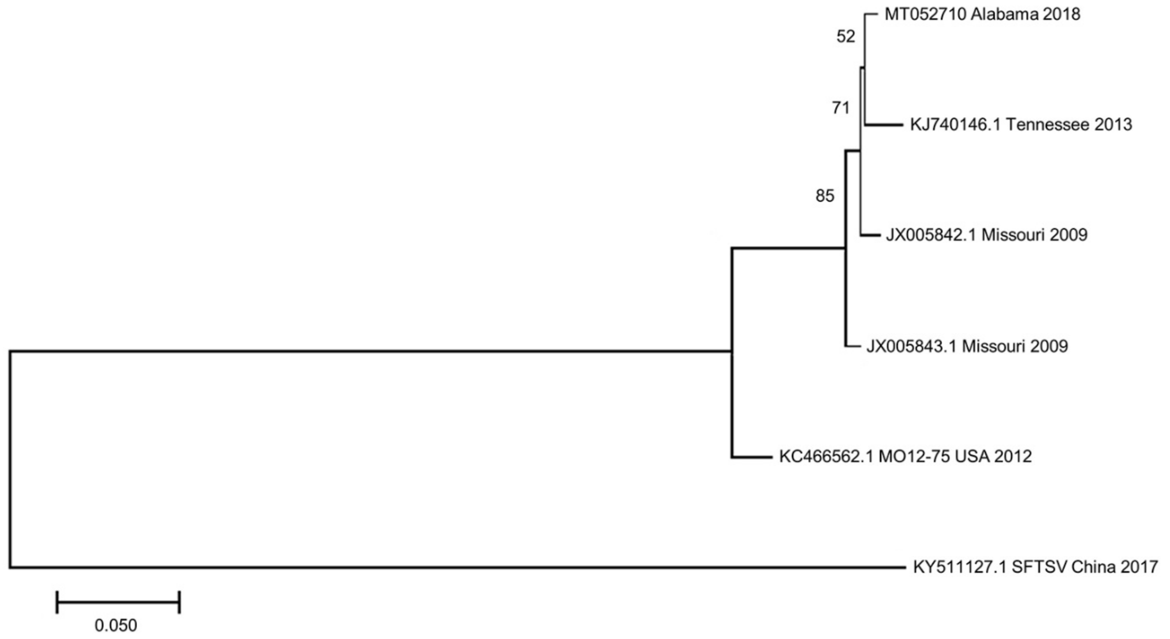


**Appendix Figure.** Maximum-likelihood phylogenetic tree showing relationships among the Alabama Heartland virus strain, submitted under GenBank accession no. MT052710, with other Heartland virus strains and SFTSV strain available through the GenBank database. GenBank accession number,

collection location, and date are included for each viral strain. Country name is included for reference strain sequences. Numbers at the branch nodes represent bootstrap values as percentages of 1,000 replications. Scale bar indicates nucleotide substitutions per site. SFTSV, severe fever with thrombocytopenia syndrome virus.
